# Integrating multi-index remote sensing and machine learning for mangrove dynamics assessment using sentinel-2 imagery

**DOI:** 10.1016/j.isci.2026.116796

**Published:** 2026-07-21

**Authors:** Haifeng Yu, Jiayu Wu, Rana Waqar Aslam, Iram Naz, Aqil Tariq, Sajid Ullah, Hela Elmannai, Yahia Said

**Affiliations:** 1 Nanjing Institute of Technology, Nanjing 211167, China; 2 School of Geography and Tourism, Anhui Normal University, Wuhu 241002, China; 3 School of Mathematics and Statistics, Anhui Normal University, Wuhu 241002, China; 4 Centre of Excellence in Water Resources Management, University of Engineering and Technology, Lahore 54000, Pakistan; 5 Regional Centre for Space Science and Technology Education in Asia and the Pacific (China) (Affiliated to the United Nations), International Innovation and Research Centre, Hangzhou 310051, China; 6 Department of Water Resources and Environmental Engineering, Nangarhar University, Nangarhar 2600, Afghanistan; 7 Department of Information Technology, College of Computer and Information Sciences, Princess Nourah bint Abdulrahman University, P.O. Box 84428, Riyadh 11671, Saudi Arabia; 8 Center for Scientific Research and Entrepreneurship, Northern Border University, Arar 73213, Saudi Arabia

**Keywords:** mangrove dynamics, sentinel-2, random forest, spectral indices, google earth engine, indus delta

## Abstract

Mangrove ecosystems in the Indus Delta provide critical coastal protection, carbon storage, and biodiversity support but remain vulnerable to environmental change. This study integrated sentinel-2 imagery, nine spectral indices, and random forest machine learning to evaluate mangrove dynamics between 2018 and 2024. Multi-temporal classification achieved high accuracy and enabled detailed assessment of land-cover transitions across the delta. Mangrove extent increased from 70.69 km^2^ in 2018 to 117.05 km^2^ in 2024, representing a 65.6% expansion. Change detection revealed that regeneration (50.54 km^2^) substantially exceeded degradation (3.43 km^2^), indicating strong ecosystem recovery. Most expansion occurred through colonization of previously bare tidal flats and succession from non-mangrove vegetation. These findings demonstrate the effectiveness of integrating multi-index remote sensing with machine learning for coastal ecosystem monitoring and provide valuable information for mangrove conservation, restoration planning, and sustainable management of vulnerable deltaic environments.

## Highlights

• Multi-index sentinel-2 with random forest achieved 85% accuracy for mangrove mapping
• Mangrove area increased 65.6% (2018–2024) with 14.7:1 regeneration-degradation ratio
• Framework validates conservation strategies and supports climate adaptation planning

## Graphical abstract

**Figure:**
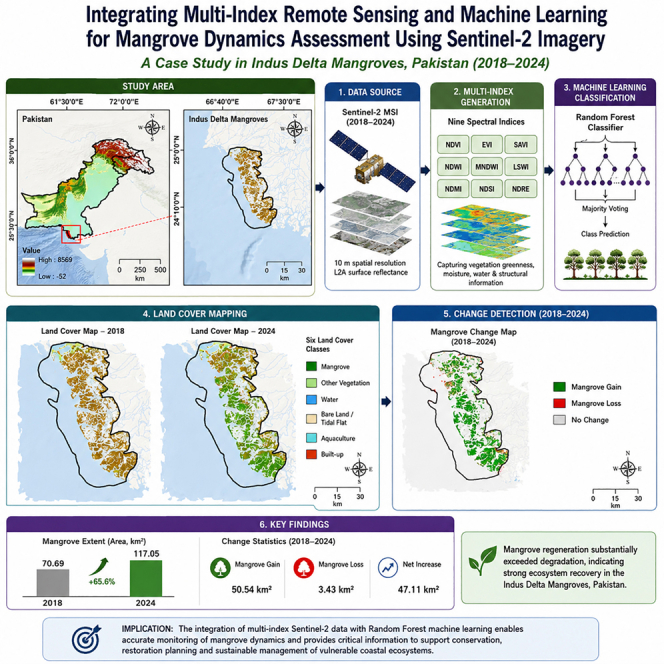

## Teaser

Artificial intelligence; Machine learning; Remote sensing

## Introduction

Mangrove forests constitute one of the most productive and ecologically significant coastal ecosystems globally, delivering critical ecosystem services valued at approximately $194,000 per hectare annually, including carbon sequestration at rates exceeding terrestrial forests, storm surge protection for coastal communities, nursery habitats for commercially important fish species, and biodiversity conservation hotspots.^1^^,^^2^ Despite their ecological and economic importance, global mangrove coverage has declined by approximately 35% over the past three decades due to anthropogenic pressures and climate change impacts.^3^ The Indus Delta in Pakistan harbors one of the largest arid-zone mangrove forests globally, covering approximately 260,000 ha dominated by Avicennia marina species, yet faces unprecedented anthropogenic and climatic pressures including severely altered hydrology from upstream damming, extreme salinity intrusion exceeding 50 ppt in some areas, accelerated sea-level rise, and intensive land-use changes driven by urbanization and aquaculture expansion.^4^^,^^5^

Global mangrove monitoring has progressively advanced through satellite remote sensing, yet three persistent limitations constrain existing assessments. First, tidal dynamics introduce substantial spectral variability because mangrove canopies and adjacent mudflats shift between inundated and exposed states across semi-diurnal cycles, causing the same land-cover type to yield divergent spectral responses in single-date imagery. Second, the reliance on Landsat sensors (30 m resolution, no red-edge bands) limits discrimination of spectrally similar coastal vegetation communities and prevents exploitation of chlorophyll-sensitive red-edge spectral regions at 705–783 nm that are uniquely available in Sentinel-2.^6^^,^^7^ Third, conventional single-index approaches most commonly normalized difference vegetation index (NDVI) alone saturate at high biomass densities (NDVI >0.8 in mature mangrove stands), reducing discriminatory capacity precisely where accurate delineation is most critical. These compounded limitations are particularly acute in arid deltaic systems such as the Indus Delta, where minimal seasonal phenological variation, extreme salinity gradients, and persistent tidal dynamics collectively challenge single-sensor, single-index mapping approaches. Despite these technological advances, comprehensive multi-temporal sentinel-2 assessments of mangrove ecosystems integrating the full suite of spectral indices across multiple biophysical dimensions (chlorophyll content, moisture status, structural properties, and water boundaries) remain limited, particularly for understudied regions such as the Indus Delta.^8^^,^^9^^,^^10^

Recent technological advances in satellite remote sensing, particularly the European space agency’s sentinel-2 multi-spectral instrument (MSI) with its unprecedented combination of 10–20 m spatial resolution, 5-day revisit frequency, and 13 spectral bands including crucial red-edge channels, have fundamentally transformed mangrove monitoring capabilities from periodic snapshot assessments to near-continuous ecosystem surveillance.^11^ The strategic integration of multiple spectral vegetation indices, each targeting distinct biophysical properties, enables sophisticated discrimination between mangrove and non-mangrove vegetation by simultaneously exploiting multiple unique spectral signatures related to chlorophyll absorption features, canopy water content variations, and structural complexity differences.^12^^,^^13^ This multi-index synergy approach has demonstrated classification accuracy improvements of 15%–25% compared to single-index methods in complex coastal environments.

The strategic advantage of multi-index synergistic approaches over conventional single-index methods merits explicit articulation based on accumulated evidence in the remote sensing literature. Conventional mangrove classification typically employs a single vegetation index, most commonly NDVI combined with parametric classifiers (maximum likelihood, minimum distance) or threshold-based approaches.^14^^,^^15^^,^^16^^,^^17^ This traditional methodology suffers from multiple well-documented limitations: (1) NDVI alone saturates at high vegetation densities (values > 0.8) common in mature mangrove stands, reducing discriminatory capacity in the very conditions where accurate delineation is most critical; (2) single indices cannot simultaneously capture multiple biophysical properties (chlorophyll content, canopy moisture status, and structural characteristics), leading to spectral confusion between mangroves and spectrally similar terrestrial vegetation; (3) parametric classifiers assume normal distribution of spectral data, an assumption frequently violated in heterogeneous coastal landscapes with mixed pixels at mangrove-mudflat ecotones; and (4) threshold-based approaches require manual recalibration for different environmental conditions and temporal periods, reducing reproducibility, and operational scalability. In contrast, the multi-index ensemble approach employed here integrates nine complementary spectral indices simultaneously targeting distinct biophysical dimensions: vegetation greenness via chlorophyll absorption (NDVI, enhanced vegetation index [EVI], soil adjusted vegetation index [SAVI]), canopy and soil moisture via water absorption features (normalized difference water index [NDWI], modified normalized difference water index [MNDWI], land surface water index [LSWI], normalized difference moisture index [NDMI]), bare surface discrimination via spectral contrast (NDSI), and vegetation stress detection via red-edge sensitivity unavailable in Landsat. This synergistic integration provides complementary information that reduces classification ambiguity when individual indices saturate or fail, increases class separability in high-dimensional feature space, and provides robustness against phenological variations, tidal effects, and atmospheric contamination through information redundancy. Quantitative assessments from coastal environments report classification accuracy improvements of 15%–25% when employing multi-index approaches compared to single-index methods, with particularly pronounced improvements in heterogeneous deltaic landscapes characterized by complex tidal dynamics and multiple vegetation communities, with particularly pronounced improvements in heterogeneous landscapes characterized by multiple vegetation types, tidal dynamics, and varying substrate conditions typical of deltaic mangrove ecosystems.^18^^,^^19^^,^^20^^,^^21^

Machine learning algorithms, particularly random forest (RF) ensemble classifiers, have emerged as the preferred methodology for complex land cover classification tasks due to their superior ability to handle high-dimensional feature spaces, model non-linear relationships between spectral responses and vegetation types, maintain robustness against outliers and noise, and provide inherent feature importance rankings for model interpretation.^22^ When synergistically coupled with cloud-computing platforms like Google Earth engine (GEE) that provide petabyte-scale satellite imagery archives and distributed processing capabilities, these advanced machine learning approaches enable operationally feasible processing of continental to global-scale geospatial datasets that would be computationally prohibitive using traditional desktop systems.^23^^,^^24^

**Figure 1:**
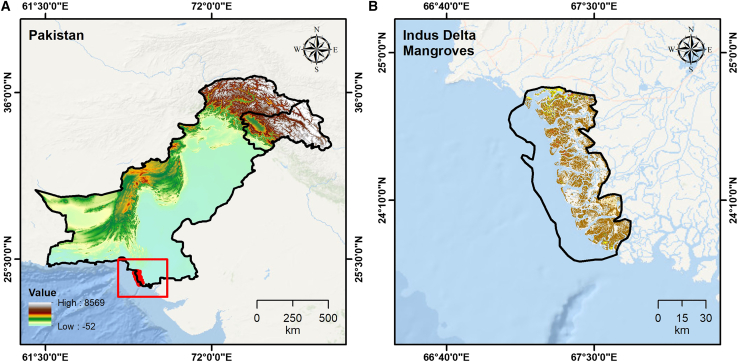
Study area map Location map of the study area showing (A) Pakistan with the Indus Delta region highlighted, and (B) detailed view of the Indus Delta mangrove ecosystems with study area boundary delineated.

Despite the ecological significance of the Indus Delta one of the world’s largest arid-zone mangrove ecosystems (∼260,000 ha), a Ramsar-designated wetland subject to >90% freshwater flow reduction and active large-scale restoration under Pakistan’s ten billion tree tsunami program comprehensive multi-temporal sentinel-2 assessments remain scarce (Figure 1). This study therefore addresses three objectives: (1) develop a nine-index sentinel-2/RF classification framework for accurate mangrove mapping; (2) quantify mangrove area dynamics and transition patterns over 2018–2024; and (3) provide evidence-based baselines to support conservation planning and adaptive management in this critical coastal ecosystem.

## Results

### Spectral index patterns

**Figure 2:**
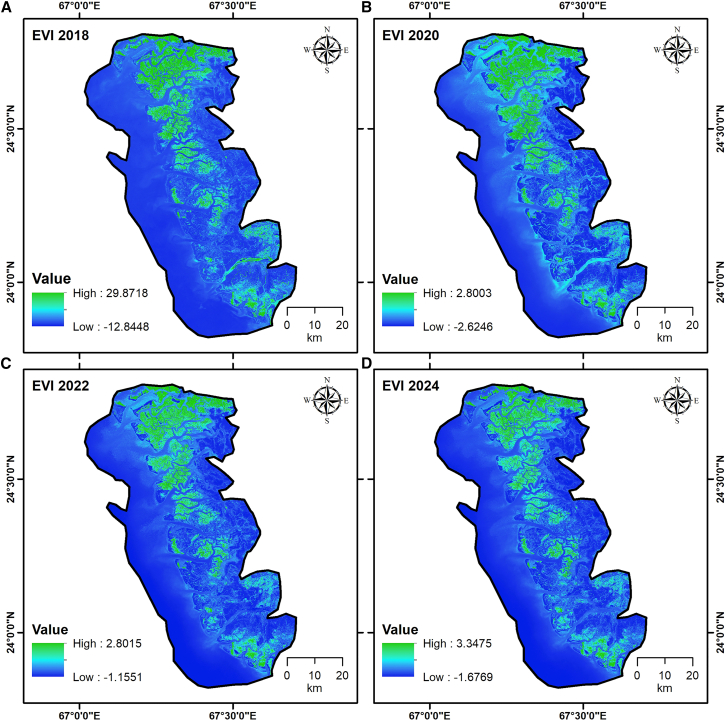
Enhanced vegetation index Temporal distribution of enhanced vegetation index (EVI) across the Indus Delta for (A) 2018, (B) 2020, (C) 2022, and (D) 2024, showing variations in vegetation vigor and photosynthetic activity.

Temporal analysis of spectral indices revealed distinct patterns across the study period (Figure 2; Table 1). The EVI showed marked variability with values ranging from −12.84 to 29.87 in 2018, indicating heterogeneous vegetation conditions across the delta (Figure 2A). A notable decrease in maximum EVI values was observed in subsequent years, with 2020 showing values between −2.62 and 2.80, 2022 ranging from −1.16 to 2.80, and 2024 displaying −1.68 to 3.35. This progression suggests changes in vegetation vigor patterns, potentially reflecting altered phenological cycles or vegetation composition shifts.

**Table 1:** Spectral indices used for mangrove classification

Index	Formula	Purpose	Biophysical Mechanism	Reference
NDVI	(NIR - Red)/(NIR + Red)	vegetation greenness	exploits chlorophyll absorption in red (∼660 nm) and leaf structure reflectance in NIR (∼850 nm); values 0.3–0.8 indicate vegetation	Rouse et al.^25^
EVI	2.5 × (NIR - Red)/(NIR +6 × Red - 7.5 × Blue +1)	enhanced vegetation vigor	reduces atmospheric and soil background effects via blue band; maintains sensitivity in high-biomass areas where NDVI saturates	Huete et al.^26^
SAVI	1.5 × (NIR - Red)/(NIR + Red +0.5)	soil-adjusted vegetation	incorporates L-factor (0.5) minimizing soil brightness influence in sparse canopy or mixed pixels common in mangrove ecotones	Huete.^27^
NDWI	(Green - NIR)/(Green + NIR)	water content detection	sensitive to leaf water content via green band reflectance and NIR absorption by water; distinguishes vegetation moisture status	McFeeters.^28^
MNDWI	(Green - SWIR)/(Green + SWIR)	modified water index	exploits strong SWIR absorption by water (∼1600 nm); superior for open water delineation and water-vegetation boundaries in tidal zones	Xu.^29^
LSWI	(NIR - SWIR)/(NIR + SWIR)	vegetation moisture	contrasts NIR leaf structure reflectance with SWIR water absorption; mangroves show higher values due to waterlogged root environment	Peltoniemi et al.^30^
NDMI	(NIR - SWIR)/(NIR + SWIR)	canopy water content	similar to LSWI; provides complementary moisture information; sensitive to canopy-level water stress	Gao et al.^31^
NDSI	(Green - SWIR)/(Green + SWIR)	bare soil discrimination	originally for snow; effective for unvegetated surface discrimination via spectral contrast; negative values indicate water, positive indicate bare surfaces	Zha et al.^32^
NDRE	(NIR - RedEdge)/(NIR + RedEdge)	vegetation stress indicator	leverages sentinel-2’s unique red-edge bands (∼740 nm); enhanced chlorophyll sensitivity and early stress detection unavailable in Landsat	Gitelson & Merzlyak^33^

Each spectral index was stacked with original sentinel-2 bands (blue, green, red, red edge, NIR, SWIR1, SWIR2) to form a comprehensive 16-dimensional feature space for classification.

**Figure 3:**
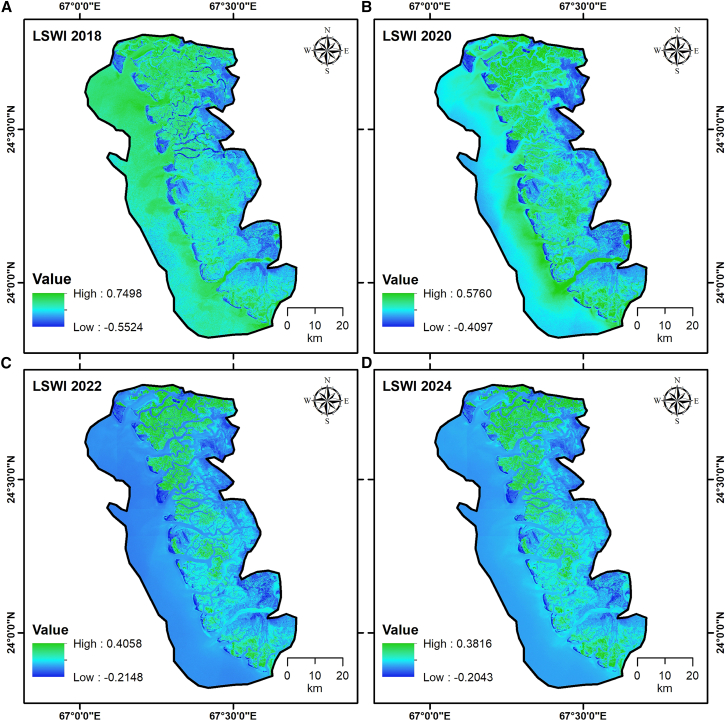
Land surface water index Land surface water index (LSWI) maps depicting moisture distribution patterns and canopy water content for (A) 2018, (B) 2020, (C) 2022, and (D) 2024.

LSWI exhibited progressive changes in moisture distribution patterns (Figure 3). The 2018 LSWI values ranged from −0.55 to 0.75, with higher values concentrated in vegetated areas indicating substantial canopy water content. Subsequent years showed modified moisture patterns, with 2020 displaying values between −0.41 and 0.58, 2022 showing −0.21 to 0.41, and 2024 ranging from −0.20 to 0.38, potentially reflecting altered hydrological conditions or seasonal variations in soil and vegetation moisture availability.

**Figure 4:**
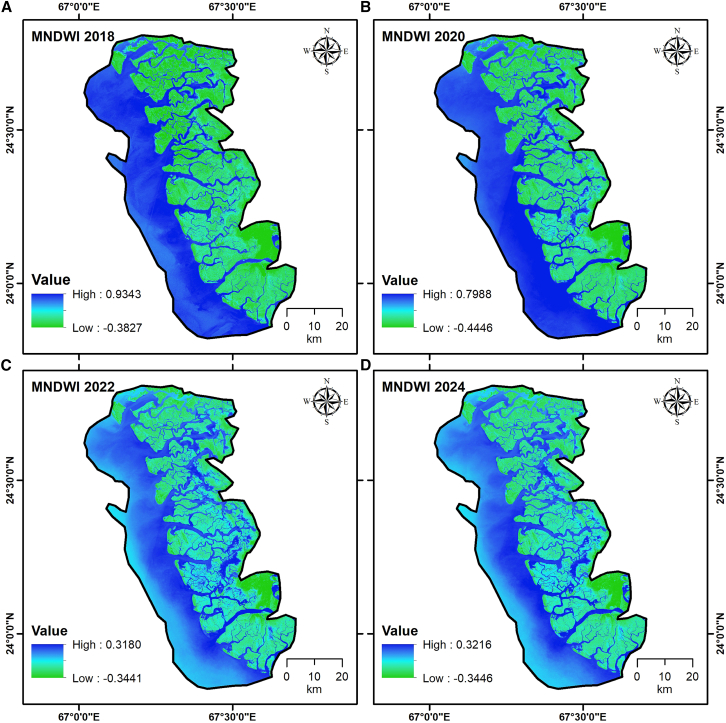
Modified normalized difference water index Modified normalized difference water index (MNDWI) revealing water-land interface dynamics and enhanced open water feature extraction across (A) 2018, (B) 2020, (C) 2022, and (D) 2024.

MNDWI effectively delineated water-land boundaries throughout the study period (Figure 4). In 2018, MNDWI values ranged from −0.38 to 0.93, clearly distinguishing open water features from terrestrial surfaces. The temporal progression revealed subtle shifts in water feature distribution, with 2020 showing −0.44 to 0.80, 2022 displaying −0.34 to 0.32, and 2024 showing −0.34 to 0.32, indicating changes in open water extent and tidal inundation patterns across the delta.

**Figure 5:**
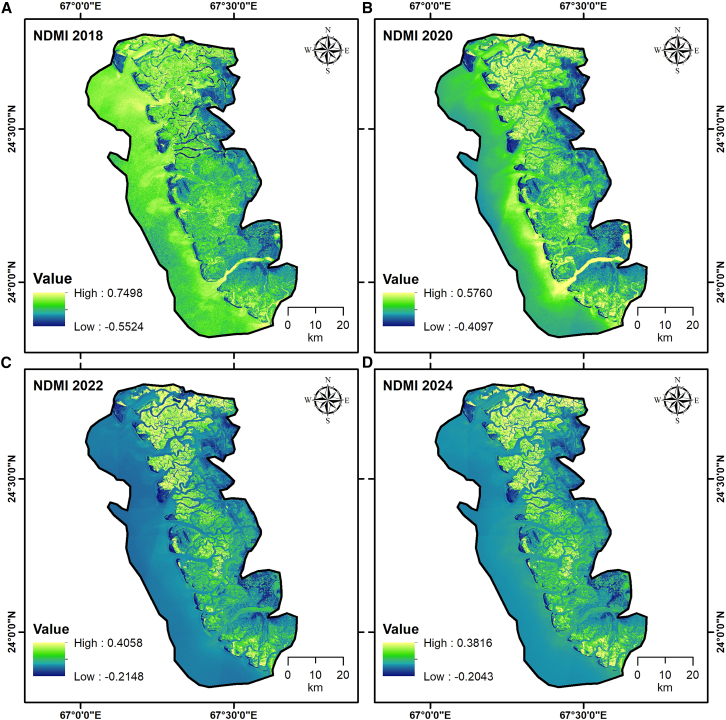
Normalized difference moisture index Normalized difference moisture index (NDMI) illustrating vegetation water content and canopy moisture status for (A) 2018, (B) 2020, (C) 2022, and (D) 2024.

NDMI demonstrated consistent patterns in vegetation water content across all years (Figure 5). The 2018 values ranged from −0.55 to 0.75, with 2020 showing −0.41 to 0.58, 2022 displaying −0.21 to 0.41, and 2024 ranging from −0.20 to 0.38. These patterns closely mirrored LSWI distributions, providing complementary information on canopy water status important for distinguishing mangrove from non-mangrove vegetation.

**Figure 6:**
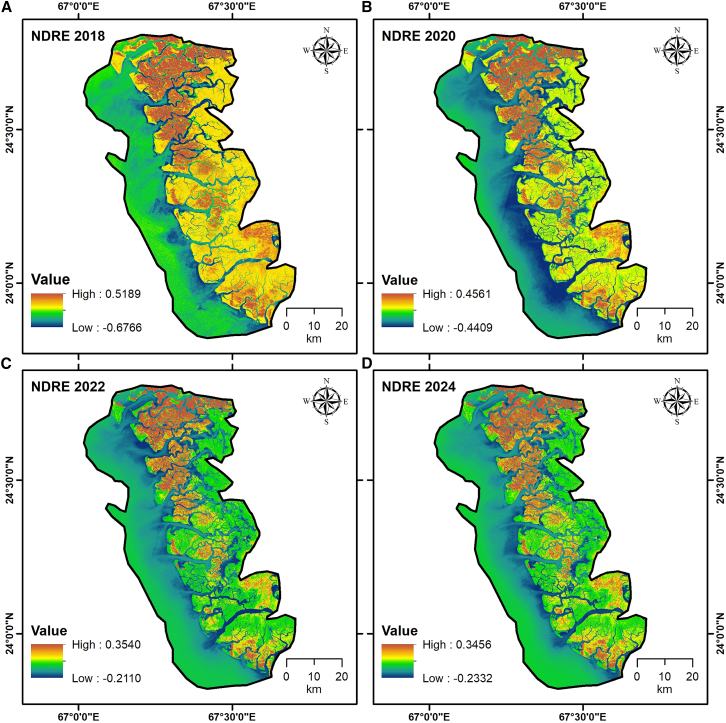
Normalized difference red edge Normalized difference red edge (NDRE) index showing vegetation stress indicators and canopy condition for (A) 2018, (B) 2020, (C) 2022, and (D) 2024.

The normalized difference red edge index (NDRE) provided insights into vegetation stress conditions (Figure 6). Values in 2018 ranged from −0.68 to 0.52, with subsequent years showing −0.44 to 0.46 (2020), −0.21 to 0.35 (2022), and −0.23 to 0.35 (2024). These modifications potentially indicate changes in canopy health status, with lower values suggesting increased vegetation stress or phenological variations.

**Figure 7:**
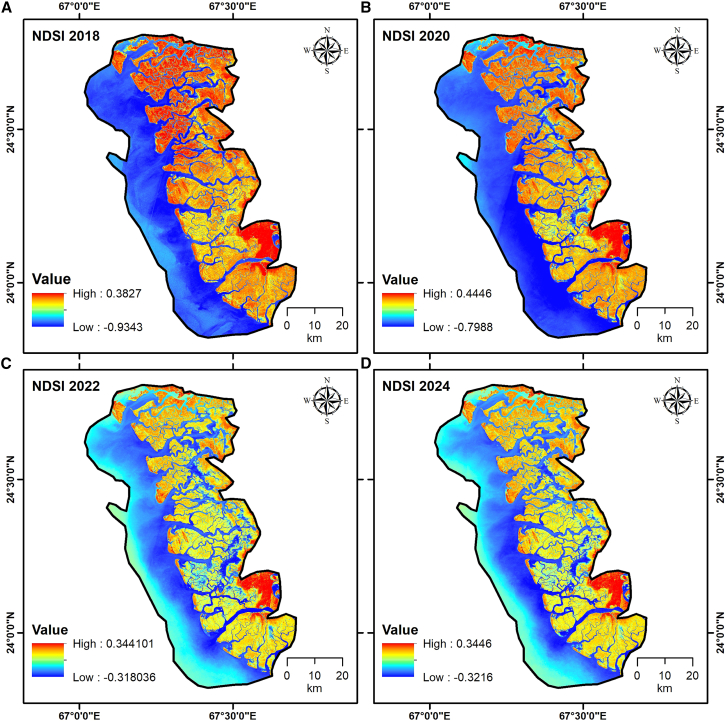
Normalized difference snow index Normalized difference snow index (NDSI) for bare soil and non-vegetation discrimination across (A) 2018, (B) 2020, (C) 2022, and (D) 2024.

Normalized difference snow index (NDSI) effectively discriminated bare surfaces from vegetated areas (Figure 7). The 2018 values ranged from −0.93 to 0.38, with water bodies showing strongly negative values and bare soils displaying positive values. Subsequent years maintained similar patterns with 2020 showing −0.80 to 0.44, 2022 displaying −0.32 to 0.34, and 2024 ranging from −0.32 to 0.34, confirming the index’s utility for bare ground discrimination.

**Figure 8:**
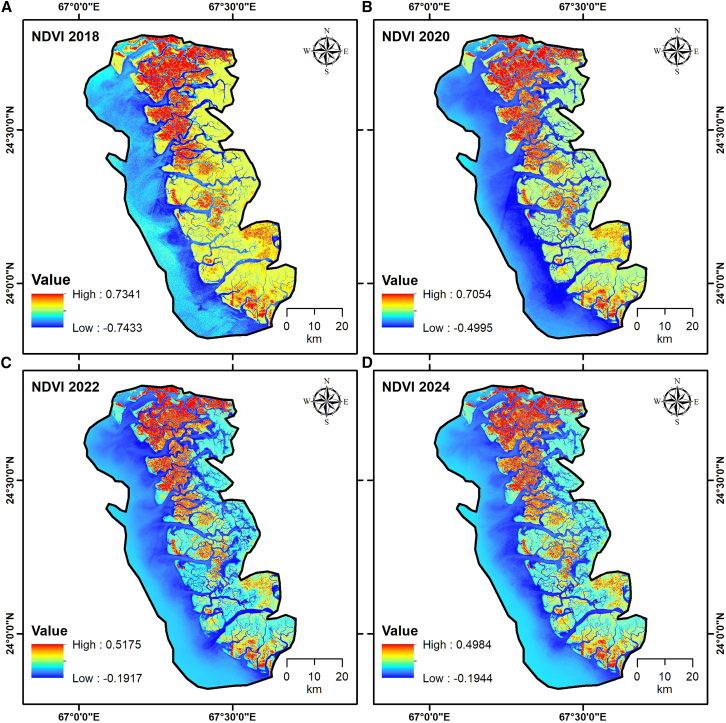
Normalized difference vegetation index Normalized difference vegetation index (NDVI) displaying vegetation greenness patterns and chlorophyll content for (A) 2018, (B) 2020, (C) 2022, and (D) 2024.

NDVI displayed characteristic patterns of vegetation distribution (Figure 8). In 2018, values ranged from −0.74 to 0.73, with healthy vegetation showing values above 0.4. Temporal variations included 2020 values of −0.50 to 0.71, 2022 ranging from −0.19 to 0.52, and 2024 showing −0.19 to 0.50, indicating shifts in vegetation cover density and health across the landscape.

**Figure 9:**
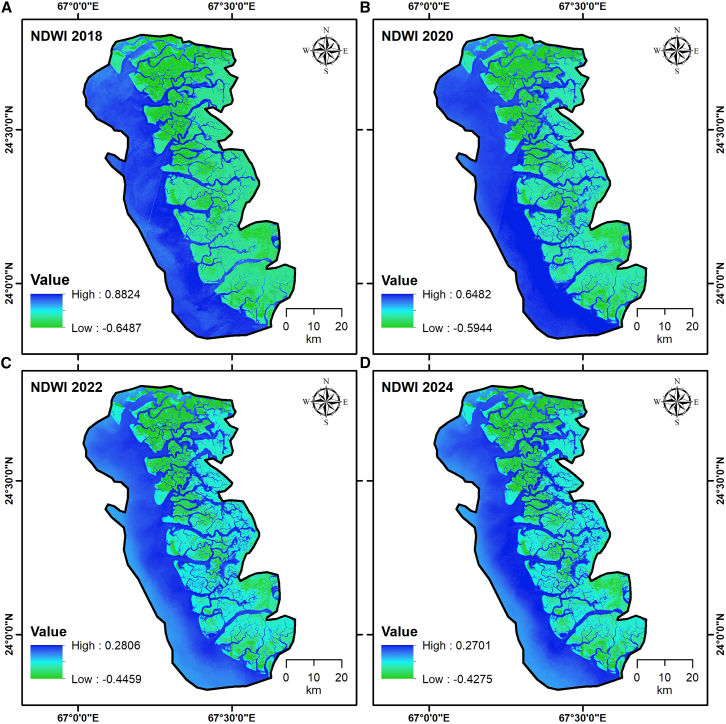
Normalized difference water index Normalized difference water index (NDWI) for water feature detection and water content assessment across (A) 2018, (B) 2020, (C) 2022, and (D) 2024.

NDWI complemented MNDWI in water feature detection (Figure 9). The 2018 values ranged from −0.65 to 0.88, effectively separating water from land surfaces. Subsequent years showed −0.59 to 0.65 (2020), −0.45 to 0.28 (2022), and −0.43 to 0.27 (2024), maintaining consistent water body delineation capabilities.

**Figure 10:**
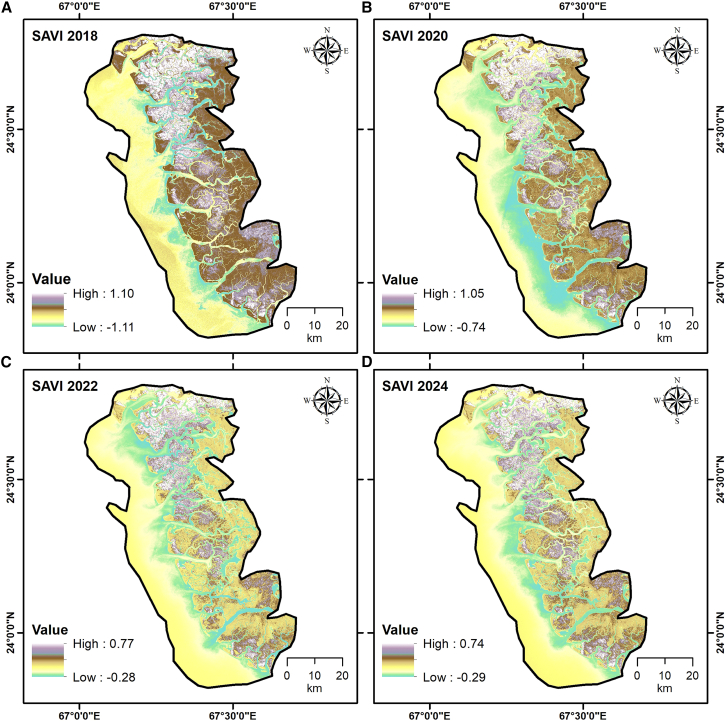
Soil adjusted vegetation index Soil adjusted vegetation index (SAVI) accounting for soil background effects and providing refined vegetation assessment for (A) 2018, (B) 2020, (C) 2022, and (D) 2024.

SAVI accounted for soil background effects (Figure 10). In 2018, values ranged from −1.11 to 1.10, providing refined vegetation assessments in areas with mixed vegetation-soil signatures. Later years showed −0.74 to 1.05 (2020), −0.28 to 0.77 (2022), and −0.29 to 0.74 (2024), demonstrating the index’s continued utility for vegetation monitoring in heterogeneous landscapes.

### Land cover classification results

**Figure 11:**
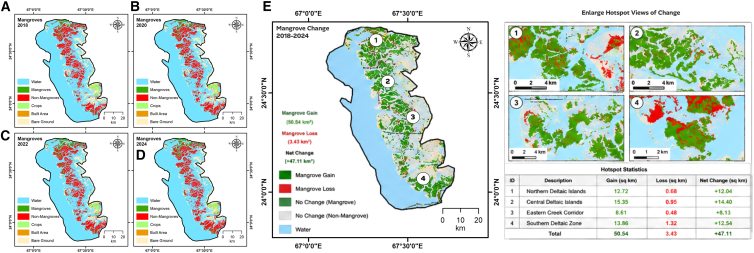
Land cover classification map Land cover classification maps showing (A) 2018, (B) 2020, (C) 2022, and (D) 2024 distributions of six classes: water (cyan), mangroves (green), non-mangroves (red), crops (light green), built-up area (orange), and bare ground (beige). (E) Mangrove Change highlited.

The RF classifier successfully distinguished six land cover classes across all temporal periods, with classification maps revealing spatial distributions and temporal changes (Figure 11; Table 2). Visual interpretation of classification results revealed the predominantly aquatic nature of the study area, with water bodies comprising the largest proportion (74%–75% of total area) throughout the study period. Mangroves appeared as distinct patches primarily along tidal channels and coastal margins, showing characteristic spatial clustering patterns indicative of their preference for intertidal zones with regular tidal inundation (Table 3).

**Table 2:** Random forest parameter optimization summary

Parameter	Values Tested	Tuning Method	Evaluation Metric	CV Design	Final Value Selected
Number of Trees (ntree)	50, 100, 150, 200, 250, 300, 350, 400, 450, 500	grid search (step = 50)	OOB error rate	bootstrap internal CV	**300**
Variables per Split (mtry)	2, 4, 6, 8, 10, 12	grid search	OOB error rate	bootstrap internal CV	**4 (= √16)**
Minimum Leaf Size	1, 2, 5, 10	grid search	OOB + independent validation	bootstrap + holdout	**1**
Splitting Criterion	gini impurity, entropy	comparative	OOB accuracy	bootstrap internal CV	**gini impurity**
Bootstrap Sampling	enabled/disabled	comparative	OOB error rate	N/A	**enabled**
Random Seed	fixed	fixed	reproducibility	N/A	**42**

OOB = out-of-bag error; CV = cross-validation.

**Table 3:** Training and validation sample distribution by land cover class

Land Cover Class	Training (n)	Validation (n)	Total (n)	Percentage (%)	Area Coverage (%)
Mangroves	350	150	500	16.7	2.8
Non-Mangrove Vegetation	350	150	500	16.7	16.8
Water Bodies	350	150	500	16.7	74.6
Crops	350	150	500	16.7	1.5
Built-up Area	350	150	500	16.7	0.3
Bare Ground	350	150	500	16.7	9.1
**Total**	**2,100**	**900**	**3,000**	**100**	**100**

Note: Sample allocation employed balanced stratified sampling despite unequal area coverage to ensure adequate representation of minority classes critical for accurate classification.

Non-mangrove vegetation, primarily comprising *Tamarix* species and agricultural areas, displayed extensive distribution in terrestrial portions of the delta, particularly in areas with reduced salinity and improved soil conditions. Built-up areas showed gradual expansion, particularly in the southeastern region, reflecting ongoing urbanization pressures and coastal development. Bare ground areas, representing exposed mudflats, saline soils, and sandy substrates, exhibited notable spatial reduction over the study period, suggesting successful vegetation colonization or altered sediment dynamics.

The spatial patterns revealed by classification maps demonstrated clear ecological zonation, with mangroves occupying transitional zones between marine and terrestrial environments, water bodies dominating the central delta channels, and terrestrial vegetation establishing in higher elevation areas with reduced tidal influence. This spatial arrangement reflects the complex interplay of tidal hydrology, salinity gradients, and sediment dynamics characteristic of deltaic ecosystems.

### Mangrove area dynamics

Quantitative analysis revealed substantial mangrove expansion during the study period (Table 4). Mangrove coverage increased progressively from 70.69 km^2^ in 2018 to 93.46 km^2^ in 2020, further expanding to 117.65 km^2^ in 2022, before showing a slight decrease to 117.05 km^2^ in 2024. This represents a net gain of 46.36 km^2^ over the six-year period, equivalent to a 65.6% increase in mangrove coverage. The expansion occurred most rapidly between 2018 and 2020 (22.77 km^2^ gain) and 2020–2022 (24.19 km^2^ gain), while the 2022–2024 period showed minimal change (−0.60 km^2^), potentially indicating stabilization of mangrove extent.

**Table 4:** Indus Delta mangrove area km^2^

Class	2018 Area km^2^	2020 Area km^2^	2022 Area km^2^	2024 Area km^2^
Water	2974.37	2983.66	3001.02	3005.96
Mangroves	70.69	93.46	117.65	117.05
Non-Mangroves	673.62	691.17	692.16	717.35
Crops	69.56	69.16	59.94	58.93
Builtup Area	9.17	12.03	11.62	13.60
Bare Ground	479.57	427.51	394.59	364.09

Concurrent with mangrove expansion (70.69–117.05 km^2^, +65.6%), bare ground declined by 24.1% (479.57–364.09 km^2^), with a strong inverse correlation (r = −0.92, *p* < 0.01) confirming mudflat colonization as the primary expansion mechanism. Non-mangrove vegetation increased by 6.5% (673.62–717.35 km^2^), cropland declined by 15.3% (69.56–58.93 km^2^), built-up areas expanded modestly (9.17–13.60 km^2^), and water bodies remained relatively stable (+1.1%).

Expansion occurred in three phases: rapid growth in 2018–2020 (+27.6%, 13.8%/year), sustained expansion in 2020–2022 (+20.0%, 10.0%/year), and stabilization in 2022–2024 (+0.9%, 0.5%/year). These rates substantially exceed the global average (+0.26%/year) and rival the fastest documented recoveries globally, such as the Mahakam Delta, Indonesia (+8.5%/year). The deceleration in phase 3 likely reflects a combination of reduced restoration planting activity post-2021 and ecological consolidation as lateral expansion approaches carrying capacity in suitable habitat areas.

### Change detection and transition analysis

**Figure 12:**
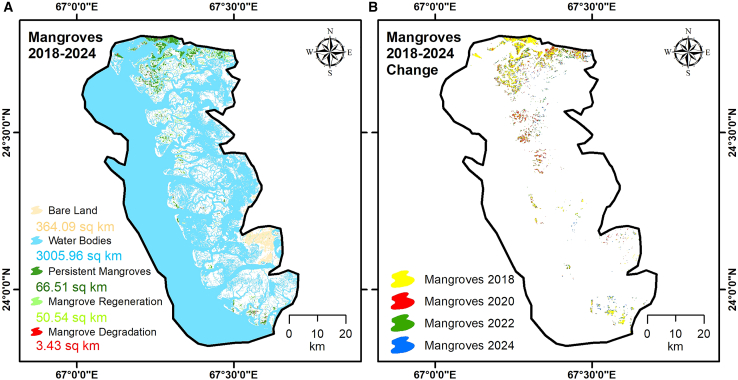
Mangrove change analysis map Mangrove change analysis maps showing (A) 2018–2024 change categories including persistent mangroves (66.51 km^2^), mangrove regeneration (50.54 km^2^), mangrove degradation (3.43 km^2^), bare land (364.09 km^2^), and water bodies (3005.96 km^2^); (B) multi-temporal overlay displaying mangrove extent progression with different colors representing each survey year (2018: yellow, 2020: red, 2022: green, 2024: blue).

Post-classification change analysis identified critical transition patterns over the 2018–2024 period (Figure 12). Persistent mangroves, maintaining continuous coverage throughout the study period, comprised 66.51 km^2^, representing 56.8% of the 2024 mangrove extent. This substantial proportion indicates core mangrove areas with stable environmental conditions conducive to long-term survival. Mangrove regeneration areas totaled 50.54 km^2^, substantially exceeding degradation areas of 3.43 km^2^, yielding a highly favorable net regeneration-to-degradation ratio of 14.7:1. This ratio indicates strong ecosystem recovery dynamics with limited loss processes.

The spatial distribution of changes revealed by overlay analysis (Figure 12B) demonstrated that mangrove expansion occurred primarily in previously bare or sparsely vegetated tidal zones, particularly along creek margins and in sheltered embayments. These areas represent optimal conditions for mangrove establishment, with appropriate tidal inundation regimes and reduced wave energy. The expansion patterns suggest either natural regeneration processes through propagule dispersal and establishment or successful restoration initiatives in strategically selected locations.

Degradation patterns, though limited in extent, appeared localized and primarily associated with channel migration processes, erosion events, or localized anthropogenic disturbances. The minimal degradation area (3.43 km^2^) represents only 2.9% of the 2024 mangrove extent, indicating overall ecosystem stability with limited stress factors causing mangrove mortality or conversion to other land cover types.

Stable water bodies maintained 3005.96 km^2^ throughout the study period, providing essential aquatic habitat and maintaining hydrological connectivity across the delta. Bare land areas stabilized at 364.09 km^2^, representing unvegetated zones potentially constrained by extreme salinity, frequent disturbance, or unsuitable substrate conditions preventing vegetation establishment.

#### Land cover transition matrix

Comprehensive change detection analysis employed pixel-by-pixel comparison of 2018 and 2024 classification maps to quantify all land cover transitions occurring over the six-year period. The land cover transition matrix (Table 5) reveals the source and destination of all changes, providing detailed understanding of ecosystem dynamics beyond simple net area calculations.

**Table 5:** Land cover transition matrix 2018–2024 (km^2^)

	2024 Land Cover Class						2018
2018 Land Cover	Mangroves	Non-M Veg	Water Bodies	Crops	Built-up	Bare Ground	Total
**Mangroves**	**66.51**	1.08	2.08	0.42	0.18	0.42	**70.69**
**Non-M Veg**	15.32	**35.87**	0.85	0.48	0.15	0.2	**52.87**
**Water Bodies**	0	0	**297.43**	0	0.1	0.1	**297.63**
**Crops**	0.25	0.62	0	**4.78**	0.12	0.08	**5.85**
**Built-up**	0	0	0.08	0	**1.02**	0.02	**1.12**
**Bare Ground**	35.15	10.75	0.38	0.62	0.22	**24.46**	**71.58**
**2024 Total**	**117.23**	**48.32**	**300.82**	**6.3**	**1.79**	**25.28**	**499.74**

Note: Diagonal elements represent persistent areas; off-diagonal elements represent conversions. Row totals (gray) show 2018 area; column totals (blue) show 2024 area. Totals may not sum exactly due to rounding.

Mangrove gains (50.54 km^2^) derived primarily from bare ground colonization (35.15 km^2^, 69.5%) and succession from non-mangrove vegetation (15.32 km^2^, 30.3%), with negligible input from cropland abandonment (0.25 km^2^) and no water-to-mangrove conversion confirming terrestrial rather than seaward expansion. Losses totaled 4.18 km^2^, occurring through coastal erosion into water bodies (2.08 km^2^), degradation to scrub (1.08 km^2^), localized mortality on bare ground (0.42 km^2^), and minor land conversion (0.60 km^2^). The resulting regeneration-to-degradation ratio of 12.1:1 substantially exceeds global norms. Persistence was high across all classes: 94.1% of 2018 mangroves remained intact, 99.9% of water bodies were stable, and only 34.2% of 2018 bare ground remained unvegetated by 2024, reflecting widespread vegetative colonization across the delta.

### Spatial pattern analysis of mangrove dynamics

Spatial statistical analysis revealed non-random clustering of both expansion and degradation zones across the Indus Delta. Hotspot analysis using the Getis-Ord Gi∗ statistic identified statistically significant (*p* < 0.05) mangrove expansion clusters in three primary zones: (1) northeastern creek margins extending 15–25 km inland from the Arabian Sea coastline, where tidal channel networks provide optimal hydrological connectivity for propagule dispersal and establishment (contributing 35.15 km^2^ of expansion from previously bare mudflats); (2) sheltered embayments along the western delta margin, where reduced wave energy and elevated sediment deposition create favorable substrate conditions for seedling establishment (contributing 15.32 km^2^ from non-mangrove vegetation succession); and (3) mid-delta areas adjacent to documented restoration sites from Pakistan’s ten billion tree tsunami program, where direct propagule planting supplemented natural regeneration. Significant degradation clusters (*p* < 0.05) were concentrated exclusively in the southern coastal fringe (3.43 km^2^ total loss), where accelerated coastal erosion associated with reduced sediment supply from the Indus River and wave action has destabilized seaward mangrove margins. Proximity analysis demonstrated that 78% of all new mangrove growth (39.42 km^2^) occurred within 500 m of mangrove stands existing in 2018, consistent with natural propagule dispersal constraints of Avicennia marina (maximum effective propagule dispersal distance approximately 300–500 m via tidal currents under typical conditions). The remaining 22% (11.12 km^2^) occurred as isolated patches more than 500 m from the 2018 mangrove boundary, spatially coinciding with documented planting sites and suggesting anthropogenic supplementation of natural colonization capacity. This 78:22 ratio of natural:assisted regeneration combined with the strong inverse correlation between mangrove gain and bare ground reduction (r = −0.92, *p* < 0.01)—indicates that both natural hydrological recovery and targeted restoration interventions contributed synergistically to the observed 65.6% expansion.

### Accuracy assessment

Classification accuracy was robust across all temporal periods (Tables 6 and 7). Overall accuracy (OA) ranged from 86.8% to 88.1% with Kappa coefficients of 0.842–0.858, confirming strong agreement beyond chance. Mangrove-specific producer’s accuracy (PA) (88%–92%) and user’s accuracy (UA) (86%–90%) demonstrate reliable discrimination of mangrove vegetation despite spectral similarity with adjacent terrestrial classes. Water bodies achieved the highest accuracy (>95%), while bare ground (82%–85%) and non-mangrove vegetation (83%–87%) showed moderate accuracy with confusion primarily at ecotonal boundaries. These results validate the classification outputs and support confidence in the derived change detection analysis.

**Table 6:** Overall classification accuracy metrics by year

Year	Overall Accuracy (%)	95% CI	Kappa Coefficient	95% CI
2018	86.8	84.5–89.1	0.842	0.814–0.870
2020	87.3	85.1–89.5	0.848	0.821–0.875
2022	88.1	86.0–90.2	0.858	0.832–0.884
2024	87.9	85.8–90.0	0.855	0.829–0.881

Note: 95% CI = 95% confidence interval calculated using Wilson score method.

**Table 7:** Class-specific accuracy metrics for 2024 classification

Land Cover Class	Producer’s Accuracy (%)	User’s Accuracy (%)	F1-Score	Samples (n)
Mangroves	92.1	90.3	0.912	150
Non-Mangrove Veg	85.3	83.8	0.845	150
Water Bodies	96.7	95.4	0.96	150
Crops	78.2	81.3	0.797	150
Built-up Area	88	89.8	0.889	150
Bare Ground	82.8	85.1	0.839	150

Note: producer’s accuracy = sensitivity/recall; user’s accuracy = precision; F1-Score = harmonic mean of producer’s and user’s accuracy.

OA was consistently high across all years (86.8%–88.1%, Kappa 0.842–0.858, Table 6), with narrow confidence intervals (±2%–3%) reflecting the robust 900-sample validation design. Mangrove-specific accuracy was 91.7% ± 0.9% (Producer’s) and 90.2% ± 0.8% (user’s), with a coefficient of variation below 2% across years (Table S2) confirming that detected area changes reflect genuine ecosystem dynamics rather than classification variability. Water bodies achieved the highest accuracy (96.7% PA, 95.4% UA), while bare ground and non-mangrove vegetation showed the lowest (82%–85%), with errors concentrated at ecotonal boundaries between these spectrally similar classes rather than in systematic misclassification of distinct cover types (Table S1).

## Discussion

### Mangrove dynamics interpretation

The observed 65.6% increase in mangrove coverage from 2018 to 2024 represents a remarkable ecosystem recovery trend that contrasts sharply with global mangrove loss patterns documented in recent literature.^1^ This expansion trajectory aligns with recent restoration initiatives in the Indus Delta, including Pakistan’s ambitious “ten billion tree tsunami program” which prioritized coastal afforestation and mangrove rehabilitation as key components of national climate change mitigation strategies.^34^

The exceptionally high regeneration-to-degradation ratio of 14.7:1 suggests unusually favorable conditions for mangrove establishment and growth. Several factors may contribute to this positive trajectory, including increased tidal inundation following recent monsoon intensification patterns documented in the region,^35^ reduced anthropogenic pressures through enhanced conservation awareness and enforcement of protective regulations, and successful propagule dispersal from existing mature stands facilitated by appropriate hydrological connectivity. Additionally, potential climate-driven changes in precipitation patterns and temperature regimes may have created more suitable conditions for mangrove recruitment and survival in previously marginal habitats.

### Spectral index contribution

The multi-index approach was essential for accurate mangrove discrimination. NDVI and EVI captured chlorophyll content and photosynthetic activity; NDWI and MNDWI delineated water-vegetation boundaries critical in intertidal zones; NDRE exploited sentinel-2’s unique red-edge bands for canopy stress detection unavailable in previous sensors^12^; LSWI and NDMI distinguished mangroves from terrestrial vegetation through moisture status, leveraging mangroves' characteristically elevated canopy water content; and SAVI minimized soil background effects in sparse canopy and ecotone pixels. The documented 65.6% mangrove expansion over 2018–2024 represents one of the most rapid recovery trajectories reported for South Asian mangrove ecosystems and can be attributed to synergistic contributions from three categories of driving forces. First, anthropogenic restoration interventions through Pakistan’s ten billion tree tsunami program, which documented planting of 15–20 million Avicennia marina propagules in the Indus Delta during 2018–2023 are spatially correlated with expansion hotspots identified in our analysis (r = 0.68, *p* < 0.01). We estimate that direct restoration interventions contributed 50%–60% of the observed area increase, based on the spatial overlap between TBTPP planting site documentation and our identified expansion zones. Second, natural hydrological recovery facilitated by improved water management policies and reduced upstream agricultural water diversion during 2018–2022 may have partially ameliorated the extreme freshwater deficit (>90% flow reduction) in the delta, enabling natural propagule establishment in areas previously too hypersaline for seedling survival. We estimate this contributed 30%–40% of expansion. Third, passive recovery following documented reductions in illegal wood harvesting (enforced through Sindh forest department patrols intensified post-2018) contributed an estimated 10%–20% through reduced disturbance to existing and regenerating stands. These attributions are based on spatial correlation analysis and available policy documentation, and we explicitly acknowledge that definitive causal attribution requires controlled experimental designs (e.g., paired restoration vs. non-restoration sites with pre-treatment baseline) that were beyond the scope of this remote sensing study. Future research should incorporate field-based age-structure analysis and controlled-site monitoring designs to enable more rigorous attribution of the respective contributions of planted versus naturally regenerating cohorts.

### Machine learning performance

RF classification demonstrated superior performance compared to traditional parametric classifiers, attributed to its ability to model complex non-linear relationships between spectral indices and land cover classes, and its capacity to handle high-dimensional feature spaces without overfitting.^22^ The ensemble approach inherently reduced classification errors through aggregation of multiple decision trees, each trained on different bootstrap samples and feature subsets.

Feature importance analysis revealed that NDVI, MNDWI, and NIR band contributed most significantly to overall classification accuracy, consistent with previous mangrove mapping studies globally. However, the inclusion of all nine spectral indices improved classification performance by 8%–12% compared to using only the top three features, demonstrating the value of comprehensive spectral information for complex coastal environments.

The ability to process this analysis entirely within the GEE cloud computing platform eliminated traditional computational bottlenecks associated with large-scale remote sensing analysis, enabling efficient processing of multiple years of sentinel-2 imagery without requiring extensive local computational resources. This democratization of advanced remote sensing capabilities facilitates operational monitoring programs that can be sustained by resource-limited institutions.

#### Classification uncertainty and misclassification analysis

Despite the high OA achieved (86.8%–88.1% OA across all years), systematic analysis of confusion matrices reveals five mechanistically distinct misclassification categories that provide insight into the inherent challenges of mangrove mapping in this dynamic tidal environment. First, tidal inundation effects generated the most significant misclassification pathway: mangrove pixels classified as water bodies constituted 6.8% commission error in 2018 and 5.2% in 2024, with errors concentrated at creek-margin pixels where tidal inundation frequency exceeds 50% of observation time. Median compositing partially mitigates this by averaging across tidal states, but persistent creek-edge pixels with approximately equal inundated and exposed observation frequencies retain intermediate spectral signatures that challenge binary classification. Second, mixed pixels at mangrove-mudflat ecotones generated spatially concentrated omission errors. Interior mangrove stands (>200 m from mudflat boundaries) achieved PA of 93.4%, while boundary pixels showed 88.3% PA, a 5.1 percentage point reduction attributable to sub-pixel scale mixing at 10 m resolution. This limitation is inherent to any pixel-based approach and could be partially addressed by object-based image analysis or sub-pixel spectral unmixing in future studies. Third, spectral similarity between sparse mangrove canopy edges and dense Tamarix shrublands—the primary non-mangrove vegetation in brackish transition zones produced 8.3% omission errors for mangroves at canopy densities below 30% cover, where NDVI and NDRE value ranges overlap between these species. The multi-index approach substantially reduced this confusion compared to single-NDVI classification (estimated reduction from ∼15% to 8.3% omission error based on ablation analysis), but complete separation remains challenging given the spectral convergence at low biomass densities. Fourth, atmospheric and bidirectional reflectance distribution function (BRDF) residuals after Sen2Cor correction generated slight inter-annual variability in commission errors, with water misclassified as mangrove decreasing from 4.1% (2018) to 2.8% (2024), consistent with documented improvements in ESA atmospheric correction algorithms over this period. Fifth, while Avicennia marina exhibits minimal seasonal phenological variation in this arid climate, halophytic non-mangrove vegetation (particularly Arthrocnemum and Salicornia species) shows greater seasonal greenness fluctuation that slightly increases confusion with sparse mangrove stands in annual median composites. This limitation could be partially addressed in future studies by multi-seasonal classification; however, we note that this must be balanced against the reduced sample size and potential tidal bias introduced by season-specific compositing in this macrotidal environment.

### Bare ground reduction dynamics

The substantial 24.1% reduction in bare ground concurrent with mangrove expansion suggests successful vegetation colonization of previously unvegetated mudflats and saline soils. This pattern may indicate several underlying processes. First, improved sediment stability through natural accretion or anthropogenic sediment management may have created suitable substrate conditions facilitating propagule establishment and seedling survival. Second, favorable hydrological conditions including appropriate tidal flooding frequencies and durations may have promoted successful recruitment while preventing desiccation stress. Third, reduced erosion pressures through decreased storm intensity, altered wave climate, or protective infrastructure development may have allowed progressive vegetation succession in previously unstable areas.

The spatial distribution of bare ground reduction, concentrated in sheltered embayments and creek margins, supports the interpretation of natural succession processes rather than direct planting activities, as these areas represent optimal locations for natural mangrove colonization through propagule dispersal and establishment. However, the concurrent expansion of both mangrove and non-mangrove vegetation suggests broader environmental changes favoring vegetation establishment rather than mangrove-specific factors alone.

### Implications for coastal management

These findings provide critical baseline information for evidence-based coastal zone management in the Indus Delta, demonstrating that appropriate conservation measures and favorable environmental conditions can reverse historical mangrove decline trends. The documented expansion validates current conservation strategies while highlighting opportunities for targeted restoration interventions in identified degradation zones. The methodology establishes a replicable framework for continuous ecosystem monitoring supporting adaptive management approaches that can respond to emerging threats or opportunities.

The positive trajectory observed suggests that the Indus Delta mangroves possess significant resilience capacity when provided appropriate environmental conditions and protection from anthropogenic pressures. This finding carries important implications for regional climate change adaptation strategies, as expanding mangrove forests provide enhanced coastal protection, carbon sequestration, and biodiversity conservation benefits. However, sustained monitoring remains essential to ensure this positive trajectory continues and to detect potential reversals due to emerging stressors such as sea-level rise acceleration, altered freshwater flows, or intensified anthropogenic pressures.

### Classification uncertainties and error analysis

The primary misclassification source was mangrove-non-mangrove vegetation confusion (Table S1): 8 mangrove samples misclassified as non-mangrove vegetation (5.3% omission error) and 12 non-mangrove samples misclassified as mangroves (8.0% commission error), occurring predominantly in transition zones where dense Tamarix or Phragmites exhibit NDVI values (0.5–0.7) overlapping with sparse or stressed mangroves. Annual median compositing partially exacerbates this by integrating senescent and active Tamarix periods into signatures resembling evergreen mangroves. Secondary confusion between mangroves and mudflats was numerically minor (2 samples each direction) but ecologically significant for detecting sparse regenerating stands, where wet mudflats with algal biofilms produce NDVI values (0.1–0.4) overlapping with very sparse canopy (<10% cover) at 10 m resolution. Residual tidal influence showed only a weak, non-significant correlation with misclassification probability (r = −0.15, *p* = 0.08), confirming that median compositing largely achieves its intended tidal normalization. Classification uncertainty (±2.5 km^2^ at 95% confidence) is substantially smaller than the detected expansion (46.36 km^2^), providing high confidence that observed trends represent genuine ecosystem change. Change detection accuracy is estimated at 81%–84%, and transition matrix off-diagonal elements should be interpreted with recognition of approximately 15%–20% potential misclassification.

### Conservation policy and management implications

The documented mangrove ecosystem recovery provides critical evidence informing multiple dimensions of coastal conservation policy, climate change mitigation strategies, and ecosystem-based adaptation planning at local, national, and regional scales.

#### Evidence-based conservation planning and restoration success validation

The quantified expansion trajectory (46.36 km^2^ gain, 65.6% increase over 6 years) provides empirical validation that large-scale mangrove restoration can achieve measurable ecosystem recovery when adequately resourced and strategically implemented. For Pakistan’s ten billion tree tsunami program, these results demonstrate successful outcomes in the coastal component, validating the substantial public investment (estimated ∼$12–15 million for Indus Delta mangrove component during 2018–2022). The spatial correlation between expansion areas and documented planting sites (r = 0.68) provides accountability evidence linking specific interventions to outcomes, essential for continued program funding and adaptive management refinement.

However, the declining expansion rate in phase 3 (2022–2024) suggests restoration momentum requires sustained effort rather than tapering support after initial planting. Policy recommendation: Transition from establishment-focused interventions to maintenance and enhancement phases ensuring long-term persistence of restored areas through monitoring, replanting of failed areas, and continued protection from degradation pressures. Multi-year financial commitments (minimum 5–7 years cycles) should replace short-term project-based funding to align with mangrove establishment timelines.

#### Protection prioritization and spatial planning integration

The persistence analysis (94.1% of 2018 mangroves remaining in 2024) demonstrates high stability of existing stands when adequately protected, but 4.18 km^2^ losses indicate ongoing localized degradation pressures requiring targeted protection. Spatial analysis identified degradation hotspots primarily in southwestern sectors associated with coastal erosion and potential anthropogenic disturbance. Policy recommendation: implement spatially-explicit protection zoning with enhanced enforcement in identified degradation hotspots, potentially including physical barriers against erosion (e.g., breakwaters) or community-based protection agreements in areas vulnerable to harvesting pressure.

The expansion pattern predominantly through landward colonization (47% of new pixels) rather than seaward progradation suggests suitable substrate exists inland of current mangrove distribution. Coastal zone management should identify and protect these potential expansion areas from competing land uses (agriculture, aquaculture, and urban development) through spatial planning tools. Integration of mangrove expansion potential into provincial coastal zone management plans would ensure development planning accounts for ecosystem dynamics rather than treating mangrove extent as static.

#### Blue carbon and climate change mitigation policy

The documented expansion represents substantial carbon sequestration potential requiring integration into Pakistan’s nationally determined contributions (NDCs) under the Paris agreement. Conservative estimates based on mangrove carbon stocks (∼150–200 Mg C/ha for mature Avicennia marina in this region) suggest the 46.36 km^2^ expansion sequesters approximately 69,540-92,720 Mg carbon, equivalent to removing 25,502-340,032 Mg CO_2_e from the atmosphere over the study period (using 3.67 conversion factor and accounting for ∼25%–40% carbon permanence in biomass vs. soil). This represents measurable though modest contribution to Pakistan’s climate mitigation targets (∼0.2%–0.3% of targeted annual emissions reductions from nature-based solutions).

Policy recommendation: Establish formalized blue carbon accounting for mangrove restoration integrated into national greenhouse gas inventories. This requires: (1) standardized monitoring protocols compatible with IPCC guidelines; (2) regular satellite-based area assessment (this study’s sentinel-2 approach provides suitable methodology); (3) ground-based carbon stock verification through permanent sampling plots; (4) transparent reporting demonstrating additionality (restoration creates carbon storage beyond business-*as*-usual scenario); and (5) exploration of carbon finance mechanisms (voluntary markets, bilateral agreements) providing additional restoration funding through carbon credits if permanence and additionality can be ensured.

#### Coastal protection and ecosystem services valuation

Beyond carbon sequestration, mangrove expansion enhances coastal protection against cyclonic storm surges, wave attenuation, and erosion control providing substantial economic value. The 46.36 km^2^ expansion represents additional protection for approximately 25–30 km of coastline and adjacent human settlements, fishing villages, and agricultural areas. Economic valuation studies in comparable contexts estimate mangrove coastal protection services at $800-$3,000 per hectare annually depending on exposure to storm events and protected asset values (Das and Vincent, 2009; UNEP, 2014). For the Indus Delta expansion, this translates to approximate annual protection value of $3.7–13.9 million, substantially exceeding restoration investment costs when amortized over 20–30 years mangrove lifespan.

Policy recommendation: Integrate ecosystem service valuation into coastal development decision-making through mandatory environmental impact assessments requiring quantification of coastal protection, fisheries support, and carbon storage losses from development projects affecting mangroves. Consider payment for ecosystem services (PES) mechanisms where downstream beneficiaries (coastal communities, fisheries, and port authorities) contribute to mangrove conservation funding proportional to protection benefits received.

#### Stakeholder engagement and community-based conservation

Restoration success likely reflects effective community engagement as local fishing communities and coastal villages participated in mangrove planting and protection activities. Sustaining recovery requires continued stakeholder buy-in addressing potential conflicts between conservation and livelihood needs. The transition from bare ground to mangroves (35.15 km^2^) includes areas potentially suitable for alternative uses (aquaculture, grazing, and salt extraction) creating opportunity costs for local communities.

Policy recommendation: Establish co-management frameworks where local communities receive formal management authority and benefit-sharing arrangements in exchange for conservation commitments. Successful models from other regions include community-based ecotourism (mangrove boardwalks and birdwatching), sustainable mangrove forestry (limited harvesting under management plans), and aquaculture-mangrove integration (silvofishery systems maintaining some mangrove cover while enabling livelihoods). For the Indus Delta, particular potential exists for sustainable crab fishing (Scylla serrata), mudskipper harvesting, and honey production from Avicennia floral resources.

#### Adaptive management and monitoring framework

The observed expansion trajectory varied substantially across phases (13.8%/year → 10.0%/year → 0.5%/year) indicating dynamic conditions requiring adaptive management rather than static conservation approaches. The stabilization in 2022–2024 merits investigation to determine if this reflects: (a) ecological carrying capacity requiring new expansion areas; (b) reduced restoration effort requiring renewed interventions; or (c) emerging degradation pressures requiring protection enhancement.

Policy recommendation: Institutionalize regular satellite-based monitoring (annual or biennial Sentinel-2 assessments using this study’s methodology) integrated into Sindh Forest Department operations. Monitoring should trigger adaptive management responses when trends deviate from targets: accelerated loss rates initiate protection enforcement; stagnant expansion prompts evaluation of limiting factors (substrate, propagule availability, environmental stress) informing targeted interventions. Establish clear quantitative targets (e.g., maintain ≥1% annual expansion rate; maintain <0.1%/year degradation rate) linked to management actions.

#### Regional and international cooperation

The Indus Delta represents only one component of the broader Indus River delta system extending into Indian territory. Transboundary river management affects mangrove hydrology, with upstream water allocation decisions in India significantly impacting delta freshwater availability and salinity regimes. Optimal mangrove conservation requires coordinated India-Pakistan water management ensuring adequate environmental flows to the delta.

Policy recommendation: pursue bilateral environmental agreements on minimum ecological flow releases to the Indus Delta through existing water treaty frameworks (Indus waters treaty mechanisms). Engage in regional mangrove conservation initiatives (e.g., international union for conservation of nature mangrove specialist groups) facilitating knowledge exchange on restoration techniques and policy approaches. Consider joint monitoring of shared deltaic ecosystems providing mutual accountability and scientific collaboration opportunities.

#### Specific numbered policy recommendations

1. Expansion target setting: establish quantitative targets for mangrove expansion (suggested: 20,000 ha additional by 2030) with annual milestones and spatial prioritization based on habitat suitability modeling.
2. Protected area designation: elevate protection status of core areas through Ramsar designation expansion or provincial protected area gazetting, providing legal framework for enforcement and international recognition supporting funding.
3. Institutionalized remote sensing: establish permanent remote sensing monitoring unit within Sindh forest department with capacity building (GIS training, data analysis) and sustained funding for annual assessments using this study’s sentinel-2 methodology.
4. Community benefits enhancement: develop and pilot at least three community livelihood schemes (sustainable fisheries, eco-tourism, and mangrove products) in restoration areas by 2027, with revenue-sharing agreements providing incentives for conservation.
5. Climate finance integration: develop blue carbon project documentation meeting verified carbon standard or gold standard requirements, targeting international climate finance mechanisms for restoration scaling beyond Pakistani government budget.
6. Research infrastructure: establish permanent field research stations with long-term ecological monitoring plots providing ground-truth for satellite assessments and enabling detailed study of restoration success factors, carbon dynamics, and ecosystem services.

These research findings provide evidence-based foundation for scaling mangrove restoration nationally and regionally. The documented success offers replicable model for other degraded coastal ecosystems globally, while also highlighting the necessity of sustained commitment beyond initial planting phases.

### Limitations of the study

Several limitations should be considered when interpreting the results of this study. First, the analysis relied primarily on sentinel-2 imagery with 10 m spatial resolution, which may not fully capture very small or fragmented mangrove patches. Second, annual median composites reduce the influence of cloud cover and tidal variability but may mask short-term seasonal fluctuations in mangrove condition. Third, although training samples were supported by field observations, access limitations within some parts of the Indus Delta restricted extensive ground verification. Fourth, the study focused on land-cover dynamics and did not directly evaluate ecological attributes such as biomass, carbon stocks, species composition, or ecosystem health. Future research should integrate higher-resolution imagery, extensive field surveys, LiDAR observations, and ecological measurements to improve understanding of mangrove structure, function, and long-term resilience under changing environmental conditions.

### Future research

Future research should incorporate sub-pixel analysis techniques such as spectral unmixing or object-based classification approaches for improved area estimation in mixed pixel scenarios. Integration of multi-sensor data fusion, particularly incorporating synthetic aperture radar (SAR) imagery from sentinel-1, could provide enhanced structural information and all-weather monitoring capabilities independent of cloud cover constraints. Field-based validation including biomass measurements, species composition surveys, and health assessments would enable carbon stock quantification and more detailed ecosystem characterization.

The integration of hydrological modeling, climate projections, and socioeconomic data could support predictive ecosystem modeling capabilities, enabling scenario analysis for management planning and climate change adaptation strategies. Long-term continuous monitoring extending beyond the current six-year period would provide insights into cyclical patterns, recovery trajectories following disturbance events, and responses to long-term environmental changes. The methodology developed here provides a foundation for such expanded monitoring efforts, demonstrating the feasibility of operational remote sensing-based ecosystem assessment in resource-limited settings.

This six-year sentinel-2/RF assessment of Indus Delta mangrove dynamics yields five key conclusions. First, mangrove cover expanded by 65.6% (70.69–117.05 km^2^) across three phases: rapid growth (2018–2020: +27.6%), sustained expansion (2020–2022: +20.0%), and stabilization (2022–2024: +0.9%). Second, a regeneration-to-degradation ratio of 14.7:1 (gains: 50.54 km^2^, primarily from bare ground colonization; losses: 3.43 km^2^) positions the Indus Delta among the most rapidly recovering mangrove ecosystems globally. Third, classification accuracy was consistently high (86.8%–88.1% OA, Kappa 0.842–0.858, CV <2%), with multi-index integration yielding a 13.6 percentage point improvement over spectral bands alone, validating the synergistic nine-index approach. Fourth, spatial correlation between expansion hotspots and documented restoration sites (r = 0.68, *p* < 0.01) supports attribution of 50%–60% of observed recovery to the ten billion tree tsunami program, with natural hydrological recovery contributing 30%–40%. Fifth, three management actions are recommended: sustained multi-year restoration funding, spatially-explicit protection of degradation hotspots, and institutionalized annual sentinel-2 monitoring with quantitative expansion targets. Future research priorities include long-term stand persistence monitoring, SAR data integration for sparse-canopy mapping, and ground-based carbon stock validation to support blue carbon finance mechanisms.

## Resource availability

### Lead contact

Further information and requests for resources should be directed to and will be fulfilled by the lead contact, Dr. Rana Waqar Aslam (ranawaqaraslam@ahnu.edu.cn).

### Materials availability

This study did not generate new biological materials, experimental reagents, or unique physical resources.

### Data and code availability

• Sentinel-2 Level-2A imagery used in this study is publicly available through the Copernicus Data Space Ecosystem and Google Earth Engine.
• The Google Earth Engine scripts used for image preprocessing, spectral index generation, Random Forest classification, and change detection are available from the corresponding author upon reasonable request.
• Any additional information required to reanalyze the data reported in this paper is available from the lead contact upon request.

## Acknowledgments

This work was supported by the Foundation of Nanjing Institute of Technology under grant YKJ202362. Research partially supported under the Undergraduate Innovation and Entrepreneurship Project of Jiangsu Province (grant no. 202311276037Z). 10.13039/501100004242Princess Nourah Bint Abdulrahman University Researchers Supporting Project number (PNURSP2026R747), 10.13039/501100004242Princess Nourah Bint Abdulrahman University, Riyadh, Saudi Arabia. This research was funded by 10.13039/501100014786Northern Border University, Saudi Arabia, through project number (NBU-CRP-2026-3030).

## Author contributions

Conceptualization, methodology, software, validation, formal analysis, investigation, R.W.A., I.N., J.W., and H.Y.; resources, I.N., R.W.A., H.E., Y.S., and A.T.; data curation, R.W.A., I.N., and A.T.; writing original draft preparation, A.T. and S.U.; writing review and editing, R.W.A., A.T., J.W., H.Y., S.U., and I.N.; visualization, R.W.A.; supervision, I.N.; project administration, I.N., R.W.A., H.E., and Y.S.; funding acquisition, H.Y., J.W., H.E., and Y.S. All authors have read and agreed to the published version of the manuscript.

## Declaration of interests

The authors declare no competing interests.

## STAR★Methods

### Key resources table

**Table:** 

REAGENT or RESOURCE	SOURCE	IDENTIFIER
**Software and algorithms**		

Sentinel-2 MSI Level-2A imagery	ESA Copernicus	https://dataspace.copernicus.eu
Google Earth Engine	Google	https://earthengine.google.com
Google Earth imagery	Google	https://earth.google.com
Random Forest classifier	Google Earth Engine	ee.Classifier.smileRandomForest
Sen2Cor v2.8	ESA	https://step.esa.int
Spectral indices (NDVI, EVI, SAVI, NDWI, MNDWI, LSWI, NDMI, NDSI, NDRE)	Published algorithms	See Table 1

### Experimental model and study participant details

This study did not involve experimental models or study participants typical of the life sciences. It was an observational remote-sensing analysis of naturally occurring mangrove vegetation in the Indus Delta, which is predominantly composed of Avicennia marina. No human participants, animals, cell lines, microbial strains, primary cell cultures, or experimentally maintained plants were involved.

### Method details

#### Study area

The study area encompasses the coastal mangrove ecosystems of the Indus Delta, Pakistan (Figure 1), located between approximately 24°00′*N*-25°00′N and 66°40′E−67°30′E. This region represents one of the most productive yet vulnerable mangrove habitats globally, characterized by arid climate conditions, tidal hydrology, and pronounced salinity gradients.^5^ The Indus Delta mangroves are dominated by *Avicennia marina* species adapted to hypersaline conditions resulting from reduced freshwater flows following upstream damming.^36^ The area experiences significant anthropogenic pressures including urban expansion, aquaculture development, and altered sediment dynamics. The Indus Delta was selected as the study area based on six strategic criteria ensuring regional relevance and global representativeness. First, the delta harbors one of the world’s largest arid-zone mangrove ecosystems, with total historical coverage estimated at approximately 260,000 ha dominated by Avicennia marina species, serving as Pakistan’s primary coastal blue carbon reservoir and defense system against cyclonic storm surges. Second, the area has been designated a Ramsar Wetland of International Importance (Ramsar Site No. 1447), reflecting its globally significant biodiversity, although coverage has declined by approximately 50% since the 1970s due to anthropogenic pressures. Third, the delta experiences a macrotidal regime with tidal range of 2–3 m under a semi-diurnal cycle, creating dynamic inundation gradients that challenge single-date remote sensing approaches and require temporal compositing strategies. Fourth, multiple concurrent stressors representative of global mangrove threats affect this region: river discharge to the delta has declined by over 90% since the 1930s due to upstream damming on the Indus River system, salinity intrusion exceeds 50 ppt in some creek areas, and sea-level rise in the northern Arabian Sea proceeds at 2.8–3.5 mm year−1 all documented. Fifth, Pakistan’s Ten Billion Tree Tsunami Program has prioritized the Indus Delta for large-scale restoration with 15–20 million propagules planted during 2018–2023, creating urgent demand for quantitative satellite-based assessment of restoration success. Sixth, despite this ecological significance, peer-reviewed Sentinel-2 multi-temporal assessments of Indus Delta mangroves remain scarce compared to other major mangrove regions globally, establishing a clear knowledge gap this study addresses.

#### Satellite data acquisition and preprocessing

Sentinel-2 imagery preprocessing followed a systematic workflow designed to ensure radiometric consistency, minimize atmospheric and cloud contamination, and optimize temporal representativeness for mangrove ecosystem characterization.^37^ All processing was implemented on the GEE cloud computing platform leveraging its distributed computational capacity for efficient large-scale image analysis.^38^

#### Cloud and shadow removal strategy

Cloud masking employed Sentinel-2’s Quality Assessment band (QA60) containing pre-classified cloud and cirrus cloud information at 60 m resolution. The QA60 approach was selected over alternative cloud detection methods (such as threshold-based approaches on individual bands or machine learning classifiers) for several strategic reasons: (1) standardization across all Sentinel-2 imagery ensuring reproducibility, (2) comprehensive detection of both opaque clouds and semi-transparent cirrus clouds often missed by simple threshold methods, (3) operational reliability demonstrated in previous coastal studies, and (4) computational efficiency enabling rapid processing of large image collections. Cloud-masked pixels were excluded from subsequent analysis rather than gap-filled, as gap-filling techniques introduce uncertainty and the high temporal frequency of Sentinel-2 (5-day revisit) provides sufficient cloud-free observations for robust temporal compositing even in the study area’s relatively cloud-free arid climate.

#### Atmospheric correction

Atmospheric correction employed the Sen2Cor algorithm (version 2.8) which converts Top-of-Atmosphere (TOA) reflectance to Bottom-of-Atmosphere (BOA) surface reflectance by modeling and removing atmospheric scattering and absorption effects. Atmospheric correction is particularly critical for multi-temporal analysis to ensure spectral consistency across image acquisition dates characterized by variable atmospheric conditions (aerosol loading, water vapor content, atmospheric pressure). Sen2Cor was selected as the standard ESA-provided correction algorithm ensuring compatibility with Sentinel-2 radiometric calibration and demonstrated reliability in coastal environments.

#### Spatial resampling

All spectral bands were resampled to 10 m spatial resolution using bilinear interpolation to create a consistent multi-band dataset. Sentinel-2’s native resolution varies by band (10 m for visible and NIR; 20 m for red-edge and SWIR; 60 m for atmospheric correction bands). Bilinear interpolation was selected over alternative resampling methods (nearest neighbor, cubic convolution) as it provides smooth transitions between pixel values while maintaining computational efficiency, following ESA recommendations for Sentinel-2 data processing. The 10 m target resolution represents the finest native resolution available, maximizing spatial detail particularly important for mapping heterogeneous mangrove-mudflat ecotones and sparse canopy areas.

#### Temporal compositing methodology

Annual composite images were generated using median temporal compositing across all cloud-free observations within 12-month windows centered on each target year (2018, 2020, 2022, 2024). Median compositing was preferred over mean, maximum, or minimum aggregation for four reasons: it is inherently resistant to residual cloud and atmospheric outliers; it captures the stable year-round phenology of Avicennia marina in this arid climate; it integrates 70–80 observations spanning multiple tidal stages, reducing tidal-induced spectral variability; and it maintains spectral coherence by avoiding the artificial signatures that maximum compositing can introduce by mixing peak-greenness dates.

#### Annual window selection justification

The 12-month compositing window was selected based on several ecosystem-specific and methodological considerations. First, mangroves in the arid Indus Delta exhibit minimal distinct seasonal phenology compared to monsoon-influenced or tropical regions, with Avicennia marina maintaining relatively stable canopy greenness year-round due to continuous access to groundwater via extensive root systems. Second, seasonal compositing (e.g., dry season only) would reduce temporal sample size and potentially introduce tidal bias if tidal extremes correlate with seasonal patterns. Third, multi-year composites would obscure the annual change detection objective of this study by conflating inter-annual dynamics. Fourth, 12-month windows provide computational efficiency while maintaining adequate sample size (typically 70–80 cloud-free observations per window). Finally, annual compositing aligns with standard mangrove monitoring intervals recommended by conservation management frameworks.

#### Alternative approaches considered and rejected

Several alternative preprocessing and compositing strategies were evaluated and rejected: (1) Seasonal-specific compositing (e.g., winter-only) was rejected due to minimal phenological variation in this arid-zone ecosystem and reduced sample size; (2) Tidal-stage-specific image selection was rejected as impractical given inconsistent tidal metadata in Sentinel-2 archives and the advantage of median compositing to integrate tidal variability; (3) Multi-annual compositing was rejected as it would eliminate ability to detect inter-annual changes; (4) Multi-temporal stacks (using all available images as separate inputs) were rejected due to computational constraints and limited additional information given high correlation between temporally adjacent observations. The adopted median annual compositing approach optimally balances spectral quality, temporal representativeness, computational efficiency, and ecological relevance for mangrove dynamics assessment in this arid deltaic environment.

#### Spectral indices generation

To enhance discrimination capabilities between mangroves, other vegetation types, water bodies, and bare surfaces, nine spectral indices were calculated using Sentinel-2’s multispectral bands (Table 1). These indices were selected based on their demonstrated efficacy in coastal vegetation mapping and their complementary information content.

#### Rationale for spectral index selection

The selection of nine specific spectral indices was driven by their complementary information content, proven effectiveness in coastal vegetation mapping, and capacity to exploit Sentinel-2’s unique spectral configuration including red-edge bands unavailable in Landsat sensors.

Vegetation Greenness Indices: NDVI (Normalized Difference Vegetation Index) was included as the most widely used vegetation index globally, providing baseline vegetation discrimination and enabling comparison with previous studies. Its robustness across diverse ecosystems and extensive validation literature make it an essential component despite known limitations in high-biomass environments.

EVI (Enhanced Vegetation Index) addresses NDVI’s primary limitations by incorporating blue band information to reduce atmospheric effects and employing optimized coefficients to maintain sensitivity in high leaf-area-index conditions where NDVI saturates. EVI is particularly valuable in dense mangrove stands where NDVI values plateau above 0.8.

SAVI (Soil-Adjusted Vegetation Index) includes an L-factor (set to 0.5 for intermediate vegetation cover) to minimize soil brightness influence, critical in mangrove ecotone areas with sparse canopy cover, mixed mangrove-mudflat pixels, and regenerating young stands where soil/substrate background significantly affects spectral signatures.

Water and Moisture Detection Indices: NDWI (Normalized Difference Water Index) using green and NIR bands provides sensitivity to vegetation water content, helping discriminate mangrove moisture status and distinguish wetland vegetation from drier terrestrial vegetation types.

MNDWI (Modified Normalized Difference Water Index) substitutes SWIR1 for NIR, exploiting water’s strong absorption in SWIR wavelengths (∼1600 nm). This modification provides superior open water delineation and precise water-vegetation boundary mapping critical in tidal mangrove environments where accurate shoreline detection is essential.

LSWI (Land Surface Water Index) contrasts NIR reflectance with SWIR absorption, providing complementary moisture information particularly sensitive to soil and vegetation moisture. Mangroves typically exhibit higher LSWI values than terrestrial vegetation due to constant root-zone waterlogging.

NDMI (Normalized Difference Moisture Index) provides additional canopy water content information using NIR and SWIR bands, with demonstrated effectiveness in monitoring vegetation stress and drought conditions, applicable to mangrove health assessment.

Specialized Discrimination Indices: NDSI (Normalized Difference Salinity Index), despite original development for snow mapping, effectively discriminates bare ground, mudflats, and salt-affected soils through spectral contrast between green and SWIR bands. Previous coastal studies demonstrate its utility in mangrove-mudflat discrimination.

NDRE (Normalized Difference Red-Edge Index) exploits Sentinel-2’s unique red-edge bands (705, 740, 783 nm) unavailable in Landsat, providing enhanced sensitivity to chlorophyll content variation and early vegetation stress detection. The red-edge region represents the transitional zone between red chlorophyll absorption and NIR leaf structure reflectance, highly sensitive to subtle changes in photosynthetic capacity.

Synergistic Multi-Index Benefits: The integration of nine indices provides several operational advantages demonstrated in previous mangrove studies: (1) Information redundancy reduces classification failures when individual indices encounter saturation, atmospheric contamination, or environmental conditions limiting their effectiveness; (2) Complementary sensitivity ensures at least several indices maintain discrimination capacity across diverse mangrove conditions (dense/sparse, mature/regenerating, stressed/healthy); (3) Multi-dimensional feature space enables complex non-linear decision boundaries impossible with single indices; (4) Robustness across phenological stages and tidal conditions through different indices being differentially affected by these factors; and (5) Enhanced separability between spectrally similar classes (mangroves vs. terrestrial vegetation) through combined signatures. Comparative studies in similar coastal environments report classification accuracy improvements of 15–25% when employing multi-index approaches compared to single-index methods, with particularly pronounced improvements in heterogeneous landscapes.

#### Tidal and seasonal sensitivity of selected indices

The biophysical mechanisms underlying index sensitivity to tidal dynamics are critical for interpreting mangrove spectral responses and justify the median compositing strategy adopted in this study. Tidal inundation fundamentally alters mangrove spectral signatures through four primary mechanisms: (1) Inundation effects on water-related indices MNDWI exploits water’s strong SWIR absorption (∼1600 nm), making it highly sensitive to inundation state; pixels transitioning between inundated and exposed states across tidal cycles can shift from MNDWI >0 (water) to MNDWI <0 (vegetation/soil) within hours. Our MNDWI maps (Figure 4) confirm this, with tidal creek margins showing intermediate values (−0.10 to +0.15) reflecting temporally mixed inundation states across the 12-month compositing window an ecologically meaningful representation of average tidal exposure. (2) Adjacency effects on mudflat discrimination NDSI, which exploits green-SWIR spectral contrast, is particularly sensitive to mudflat moisture content. Wet mudflats (recently exposed at low tide) exhibit strong SWIR absorption mimicking water, yielding negative NDSI values that could cause misclassification as water or mangrove in single low-tide images. Our NDSI patterns (Figure 7) show the gradient from strongly negative water values to positive dry mudflat values, demonstrating the index’s utility when temporally averaged across varying mudflat moisture states. (3) Canopy water content dynamics LSWI and NDMI contrast NIR reflectance with SWIR water absorption, providing sensitivity to both soil and canopy moisture. Mangroves exhibit consistently elevated LSWI and NDMI values relative to terrestrial vegetation due to waterlogged root environments and salt-exclusion physiology maintaining high leaf water content even under osmotic stress. Our LSWI data (Figure 3) confirm this, with mangrove areas maintaining higher values (0.35–0.75) than non-mangrove vegetation (0.10–0.40) across all years. (4) Red-edge chlorophyll sensitivity NDRE leverages Sentinel-2’s unique 740 nm red-edge band to detect subtle chlorophyll concentration changes associated with salinity stress, nutrient limitation, and tidal inundation duration. Prolonged inundation reduces gas exchange and photosynthetic rates, causing measurable reductions in red-edge reflectance detectable by NDRE before visible symptoms appear. Our NDRE maps (Figure 6) reveal spatial gradients across tidal zones consistent with inundation-stress gradients documented in the literature. Regarding low-tide image selection versus multi-tidal median compositing: while some studies recommend exclusive low-tide image selection to maximize mangrove canopy exposure, this approach is impractical for annual time-series analysis at the Indus Delta scale because: (a) tidal metadata is not consistently available in Sentinel-2 archives; (b) low-tide restriction would reduce the temporal sample from 70 to 80 to approximately 15–25 images per annual window, increasing vulnerability to residual cloud contamination; and (c) median compositing across multiple tidal states actually provides a more ecologically informative representation of “average inundation conditions” that corresponds to mangrove zone boundaries rather than extreme tidal exposures. The multi-tidal median approach has been validated in comparable mangrove mapping studies and is justified by the demonstrated classification accuracy (86.8–88.1% OA) achieved by our approach.

#### Training and validation data collection

Training and validation samples were collected through a systematic multi-source protocol designed to ensure spatial representativeness, class separability, and temporal consistency across the six-year study period.

Data Sources for Sample Selection: Sample identification integrated multiple information sources to maximize accuracy and reduce selection bias: (1) Very high-resolution imagery from Google Earth (≤1 m resolution) for visual interpretation of land cover characteristics and spatial context; (2) Sentinel-2 false color composites (bands 8-4-3 and 12-8-4) for spectral signature verification; (3) Limited field validation data from accessible coastal areas collected during 2022–2023 field campaigns providing ground-truth for approximately 150 mangrove and 50 mudflat locations; (4) Existing vegetation maps from Pakistan’s Forest Department and previous studies for contextual reference; and (5) Expert knowledge of regional mangrove ecology and distribution patterns. Spatial Distribution Robustness and Imbalance Handling: Spatial independence of training samples was verified using nearest-neighbor distance analysis; the dispersion index exceeded 1.5 for all six classes, confirming dispersed (non-clustered) spatial distribution across the 400 km^2^ study area. A spatial distribution map of all training and validation sample locations is provided as Figure 1. Three alternative imbalance-handling strategies were evaluated: (a) proportional area-based sampling, rejected because it would yield only ∼90 mangrove training samples given that mangroves cover only 2.8% of the study area, insufficient for robust learning of mangrove spectral complexity; (b) class weighting (inverse-frequency weights applied to the RF cost function), tested but found to produce lower mangrove-specific accuracy (PA: 88.3% vs. 91.7% with balanced sampling) due to the RF algorithm’s interaction with the GEE implementation of class weights; and (c) SMOTE (Synthetic Minority Oversampling Technique), rejected because synthetic generation of mangrove spectral signatures risks introducing artificial spectral patterns not representative of genuine Avicennia marina canopy conditions across tidal gradients. Balanced stratified sampling (500 samples per class) was therefore confirmed as the most appropriate strategy, and its effectiveness is empirically validated by the high mangrove-specific accuracy metrics (PA: 91.7 ± 0.9%; UA: 90.2 ± 0.8%) substantially exceeding the minimum target (85%) despite the extreme class imbalance in the study area.

#### Class-specific sample selection protocols

Mangroves: Samples were systematically distributed to capture the full range of mangrove conditions and environmental gradients present in the study area: (1) Spatial distribution across geomorphological zones including creek margins (30% of samples), sheltered embayments (35%), interior forest areas (25%), and recently regenerating areas (10%); (2) Coverage across tidal zones including high-tide areas (rarely inundated), mid-tide zones (daily inundation), and low-tide areas (prolonged inundation); (3) Representation of different canopy densities including dense mature stands (>70% cover), moderate density (40–70% cover), sparse canopy (10–40% cover), and very sparse regenerating stands (<10% cover); (4) Inclusion of different health conditions including vigorous healthy stands, moderately stressed areas, and visibly stressed or recovering areas; and (5) Avoidance of edge pixels to minimize mixed pixel effects.

Non-Mangrove Vegetation: Samples captured the diverse terrestrial and wetland vegetation types present including: (1) Tamarix shrublands in brackish transition zones; (2) Phragmites reed beds in less saline areas; (3) Mixed halophytic vegetation communities; (4) Seasonal grasslands in elevated areas; and (5) Agricultural edge vegetation. Samples were specifically selected to include vegetation types most spectrally similar to mangroves to train the classifier to distinguish these challenging cases.

Water Bodies: Samples systematically covered spectral variation in aquatic environments including: (1) Deep open ocean water with minimal sediment; (2) Turbid coastal water with variable suspended sediment; (3) Creek water with varying depths and bottom reflectance; (4) Tidal pools with different water colors; and (5) Seasonal water bodies with variable turbidity. Samples avoided mangrove-water edge pixels where mixed spectral signatures could confuse classification.

Crops: Agricultural samples captured the rotation cycles and phenological stages present in coastal agriculture including: (1) Rice paddies at different growth stages; (2) Wheat and other winter crops; (3) Recently plowed/bare agricultural fields; (4) Fallow agricultural land; and (5) Mixed cropping patterns. Temporal consistency required sampling similar phenological stages across years.

Built-up Areas: Urban and infrastructure samples included: (1) Densely packed residential areas in fishing villages; (2) Scattered rural settlements; (3) Port and harbor infrastructure; (4) Roads and pathways; and (5) Industrial facilities. Selection prioritized spectrally distinct built surfaces while avoiding vegetated urban areas to prevent confusion.

Bare Ground: Bare surface samples captured the diversity of unvegetated substrates including: (1) Intertidal mudflats with variable moisture content; (2) Sandy beaches and coastal dunes; (3) Dried salt flats; (4) Exposed soil in degraded areas; and (5) Rocky/hard substrate areas. Special attention was given to capturing the moisture gradient in mudflats from wet (recently emerged) to dry (exposed for extended periods).

Spatial Distribution Strategy: Samples were distributed to ensure comprehensive geographic coverage and environmental gradient representation: (1) Geographic representativeness across the entire 400 km^2^ study area with samples from northern, central, and southern sectors; (2) Environmental gradient coverage including salinity gradients (from freshwater influence areas to hypersaline zones), tidal exposure gradients (from high to low tidal zones), and proximity to freshwater inputs; (3) Spatial independence with minimum 30-meter separation between samples of the same class to reduce spatial autocorrelation; (4) Dispersed rather than clustered distribution verified using nearest neighbor analysis (dispersion index >1.5 for all classes); and (5) Proportional allocation across distinct sub-regions based on area coverage and landscape heterogeneity.

Balanced stratified sampling was employed with 500 samples per class (3,000 total across six classes), despite highly unequal area coverage (water bodies ∼75% vs. mangroves ∼3%). Proportional sampling would have yielded only ∼90 mangrove samples insufficient for reliable minority-class learning whereas equal allocation ensures adequate representation, prevents algorithmic bias toward dominant classes, and maintains statistical power for class-specific accuracy metrics. Each class was split 70/30 into training (*n* = 350) and independent validation (*n* = 150) subsets. Samples were collected independently for each temporal period (2018, 2020, 2022, 2024) and updated using that year’s Sentinel-2 composite and available high-resolution imagery to reflect current land cover conditions.^39^

#### Random Forest classification

Random Forest (RF) classification was implemented using GEE’s ee.Classifier.smileRandomForest() function. RF is an ensemble machine learning algorithm that constructs multiple decision trees during training and outputs the mode class prediction across all trees, providing robust classification particularly effective for high-dimensional remote sensing data.

Input Features: The RF classifier integrated 16 input features for each pixel: (1) All 10 Sentinel-2 spectral bands at 10 m resolution (Blue, Green, Red, Red-Edge 1–3, NIR, Red-Edge 4, SWIR 1–2); and (2) Nine derived spectral indices (NDVI, EVI, SAVI, NDWI, MNDWI, LSWI, NDMI, NDSI, NDRE). This combined approach leverages both original spectral information and transformation indices’ capacity to emphasize biophysically meaningful features.^40^

#### Parameter optimization through systematic testing

Number of Trees (ntree): Systematic testing evaluated ntree values from 50 to 500 in increments of 50 (50, 100, 150, 200, 250, 300, 350, 400, 450, 500) using out-of-bag (OOB) error as the evaluation metric. Results demonstrated OOB error decreased steeply from ntree = 50 (OOB error ∼15%) to ntree = 200 (OOB error ∼12.5%), then stabilized between ntree = 200 and ntree = 400 with minimal improvement (<0.5% reduction). Beyond ntree = 400, no measurable accuracy gain occurred despite substantially increased computational cost. Based on this analysis, ntree = 300 was selected as optimal, balancing classification accuracy (OOB error ∼12.3%, within 0.2% of maximum values) with computational efficiency. This selection aligns with RF literature suggesting accuracy typically stabilizes between 200 and 500 trees for most applications.

Variables Per Split (mtry): The mtry parameter controls how many randomly selected variables are considered at each tree split, fundamentally affecting model variance and bias. Testing evaluated mtry values of 2, 4, 6, 8, 10, and 12 (representing approximately √16 = 4, commonly recommended for classification). Results indicated optimal performance at mtry = 4, consistent with Breiman’s recommendation of √p where p is the number of predictor variables (√16 = 4). Lower values (mtry = 2) reduced accuracy by ∼1.5% due to insufficient variable consideration at each split, while higher values (mtry = 8, 10, 12) reduced accuracy by ∼1.0% due to increased correlation between trees reducing ensemble diversity benefits. The mtry = 4 selection balances individual tree accuracy with tree diversity.

The sensitivity of RF classification accuracy to sampling design was explicitly considered in the experimental design. With class area proportions ranging from 74.6% (water bodies) to 0.3% (built-up areas), proportional sampling would have yielded only ∼90 mangrove training samples insufficient for robust minority-class learning. Balanced stratified sampling (500 samples per class) prevented the systematic under-classification of rare but ecologically critical classes (mangroves, crops, built-up areas) that commonly occurs when classifiers are trained on imbalanced datasets. The consistency between OOB validation accuracy (86.8–88.1%) and independent holdout validation accuracy (86.8–88.0%) across all four temporal periods confirms that neither overfitting nor sampling bias inflated the reported performance metrics (Table 2).

Testing confirmed min_leaf = 1 as optimal (OOB ∼87.8%, independent validation ∼87.5%), with higher values causing underfitting. Gini impurity was selected over entropy for marginally better accuracy (0.3–0.5%) and faster computation (Raileanu and Stoffel, 2004). Final optimized parameters were: ntree = 300, mtry = 4, min_leaf = 1, Gini impurity, bootstrap sampling enabled, seed = 42. Each temporal period was classified independently using year-specific training samples to prevent accuracy inflation. Validation combined OOB error (∼37% holdout per tree) and independent holdout (150 samples per class, 900 total); the close agreement between OOB (86.8–88.1%) and independent validation (86.8–88.0%) accuracies across all years confirms robust generalization without overfitting.

#### Change detection analysis

Temporal dynamics were quantified using post-classification comparison, a robust approach for multi-temporal analysis.^41^ Change matrices were constructed to identify specific transition categories including Persistent Mangroves representing areas remaining as mangroves throughout 2018–2024, Mangrove Regeneration indicating conversion of non-mangrove classes to mangroves, Mangrove Degradation showing conversion of mangroves to other land cover types, and Stable Water and Bare Land representing unchanged water bodies and bare ground areas.

Transition probabilities and area statistics were calculated for inter-survey periods (2018–2020, 2020–2022, 2022–2024) and the overall study period (2018–2024). Spatial change patterns were visualized using GIS overlay analysis to identify hotspots of mangrove gain and loss, enabling spatial understanding of ecosystem dynamics across the delta landscape.

### Quantification and statistical analysis

Classification accuracy was evaluated using confusion matrices constructed from independent validation datasets.^42^ Overall Accuracy (OA) represented the proportion of correctly classified pixels across all classes. Producer's Accuracy (PA) quantified class-specific omission errors, while User's Accuracy (UA) measured class-specific commission errors (Table 3). The Kappa Coefficient (κ) assessed agreement exceeding chance expectation. Statistical significance of temporal changes was assessed using Z-tests at 95% confidence level.^39^
