# Supplementary material for: Integrating multi-index remote sensing and machine learning for mangrove dynamics assessment using sentinel-2 imagery
Source: iScience. 2026 Jul 21;29(8):116796. doi: 10.1016/j.isci.2026.116796 (PMC13392870; doi:10.1016/j.isci.2026.116796)
Supplement: Document S1. Tables S1–S2 [file mmc1.pdf]

iScience, Volume *29*

Supplemental information

Integrating multi-index remote sensing and machine learning for mangrove dynamics assessment using sentinel-2 imagery

Haifeng Yu, Jiayu Wu, Rana Waqar Aslam, Iram Naz, Aqil Tariq, Sajid Ullah, Hela Elmannai, and Yahia Said

Supplemental Material

Table S1. Confusion matrix for 2024 classification (sample counts)

	Classified As →							
**Reference Class ↓**	**Mang**	**Non-M Veg**	**Water**	**Crops**	**Built-up**	**Bare**	**Total**	**PA(%)**
**Mangroves**	**138**	8	0	2	0	2	**150**	92
**Non-M Veg**	12	**128**	0	3	0	7	**150**	85.3
**Water Bodies**	0	0	**145**	0	2	3	**150**	96.7
**Crops**	3	5	0	**117**	8	17	**150**	78
**Built-up Area**	0	0	3	6	**132**	9	**150**	88
**Bare Ground**	2	12	4	6	2	**124**	**150**	82.7
**Total**	**155**	**153**	**152**	**134**	**144**	**162**	**900**	
**UA (%)**	**89**	**83.7**	**95.4**	**87.3**	**91.7**	**76.5**		**87.9**

Note: PA = Producer's Accuracy (recall); UA = User's Accuracy (precision); Diagonal elements represent correct classifications

Table S2. Mangrove classification accuracy across all years

**Year**	**Producer's Accuracy (%)**	**User's Accuracy (%)**	**F1-Score**	**Commission Error (%)**	**Omission Error (%)**
2018	90.7	89.3	0.9	10.7	9.3
2020	91.3	89.8	0.906	10.2	8.7
2022	92.7	91.2	0.92	8.8	7.3
2024	92.1	90.3	0.912	9.7	7.9
**Mean**	**91.7**	**90.2**	**0.91**	**9.8**	**8.3**
Stdev	0.9	0.8	0.009	0.8	0.9
CV (%)	1	0.9	1	8.2	10.8

Note: Commission Error = 100 - User's Accuracy; Omission Error = 100 - Producer's Accuracy; CV = Coefficient of Variation indicating temporal consistency
